# Rutin Linoleate Triggers Oxidative Stress-Mediated Cytoplasmic Vacuolation in Non-Small Cell Lung Cancer Cells

**DOI:** 10.3390/life14020215

**Published:** 2024-02-01

**Authors:** Iasmina Marcovici, Daliborca Vlad, Roxana Buzatu, Ramona Amina Popovici, Raluca Mioara Cosoroaba, Raul Chioibas, Andreea Geamantan, Cristina Dehelean

**Affiliations:** 1 Faculty of Pharmacy, “Victor Babes” University of Medicine and Pharmacy Timisoara, Eftimie Murgu Square No. 2, 300041 Timisoara, Romania; 2 Research Center for Pharmaco-Toxicological Evaluations, Faculty of Pharmacy, “Victor Babes” University of Medicine and Pharmacy Timisoara, Eftimie Murgu Square No. 2, 300041 Timisoara, Romania; 3 Discipline of Pharmacology, Department of Pharmacology and Biochemistry, Faculty of Medicine, “Victor Babes” University of Medicine and Pharmacy Timisoara, Eftimie Murgu Square No. 2, 300041 Timisoara, Romania; 4 Department of Dentofacial Aesthetics, Faculty of Dental Medicine, “Victor Babes” University of Medicine and Pharmacy Timisoara, 9 Revolutiei 1989 Ave., 300070 Timisoara, Romania; 5 Department of Management, Legislation and Communication in Dentistry, Faculty of Dental Medicine, “Victor Babes” University of Medicine and Pharmacy Timisoara, Eftimie Murgu Square No. 2, 300041 Timisoara, Romania; 6 Department of Surgery I, Faculty of Medicine, “Victor Babes” University of Medicine and Pharmacy Timisoara, Eftimie Murgu Square No. 2, 300041 Timisoara, Romania

**Keywords:** rutin oleate, rutin linoleate, non-small cell lung cancer, cytotoxicity, cytoplasmic vacuolation, oxidative stress

## Abstract

Lung cancer (LC) represents one of the most prevalent health issues globally and is a leading cause of tumor-related mortality. Despite being one the most attractive compounds of plant origin due to its numerous biological properties, the therapeutic applications of rutin (RUT) are limited by its disadvantageous pharmacokinetics. Thus, the present study aimed to evaluate in vitro the application of two RUT fatty acids bioconjugates, rutin oleate (RUT-O) and rutin linoleate (RUT-L), as potential improved RUT-based chemotherapeutics in non-small cell lung cancer (NSCLC) treatment. The results indicate that both compounds lacked cytotoxic potential in EpiAirway™ tissues at concentrations up to 125 µM. However, only RUT-L exerted anti-tumorigenic activity in NCI-H23 NSCLC cells after 24 h of treatment by reducing cell viability (up to 47%), proliferation, and neutral red uptake, causing cell membrane damage and lactate dehydrogenase (LDH) leakage, affecting cytoskeletal distribution, inducing cytoplasmic vacuolation, and increasing oxidative stress. The cytopathic effects triggered by RUT-L at 100 and 125 µM are indicators of a non-apoptotic cell death pathway that resembles the characteristics of paraptosis. The novel findings of this study stand as a basis for further investigations on the anti-cancer properties of RUT-L and their underlying mechanisms.

## 1. Introduction

Lung cancer (LC) stands as one of the leading causes of tumor-related deaths worldwide, rating the second most frequently diagnosed cancer type in both men and women after prostate and breast neoplasms, respectively [1]. Globally, over 1 million people die every year due to its aggressive nature [2]. LC comprises two broad classes, namely, small-cell lung carcinoma (SCLC), and non-small cell lung carcinoma (NSCLC), the latter category accounting for the majority of LC diagnoses (80–85%) [3], and is further divided into three histologic subtypes—adenocarcinoma (40%), squamous cell carcinoma (25–30%), and large cell carcinoma (10–15%) [4]. LC development is triggered by a complex interplay between genetic background (e.g., heredity, gene mutations), behavioral habits (e.g., tobacco smoking), and environmental factors (e.g., exposure to radon or asbestos, chronic infections) [4]. However, smoking remains the primary cause of LC initiation due to the umpteen carcinogens contained in cigarette smoke, as well as the production of reactive oxygen species (ROS) that consequently lead to inflammation [2]. The current treatment modalities for LC vary from surgery to radiotherapy, chemotherapy, or targeted therapy. The recommendations are highly based on several factors, such as the LC type and disease stage. Nonetheless, despite the tremendous progress achieved over the last few decades in LC diagnosis and treatment, the efficacy of the available therapies remains poor and unsatisfactory [5], thus hastening the demand for alternative options. 

Natural products have been propelled in modern research as potential multi-targeted agents in cancer treatment due to their innate cytostatic activity resulting from their ability to modulate oxidative stress, inflammation, and cell death [6]. Phytocompounds are remarked as agents with notable relevance for drug discovery and development due to their limited adverse effects, distinguishing them from conventional chemotherapeutics that cause serious toxicity during treatment (e.g., nausea and vomiting, alopecia, myelosuppression, or mucositis) [7]. Among the numerous classes of natural compounds, flavonoids, which are benzo-gamma-pyrone derivatives widely distributed in various vegetal species, are referred to as nature’s tender drugs due to their broad spectrum of biological activities, ranging from antioxidant properties to immunomodulatory, anti-microbial, anti-inflammatory, and anti-tumor effects [8]. In particular, the anticancer activity of flavonoids resides in their efficiency in inducing apoptosis or autophagy, inhibiting cell proliferation, and triggering cell cycle arrest [9]. Rutin (RUT), also referred to in the literature as sophorin or rutoside, stands out from its group as one of the most attractive phytochemicals and a strong nutraceutical owing to its multispectral health-improving properties (i.e., radical-scavenging, anticarcinogenic, cardio- and neuro-protective, etc.) [10,11]. The use of RUT has outweighed the benefits of other flavonoids owing to its non-oxidizable and non-toxic properties [8]. Structurally, RUT is a flavonol-type plant-derived compound consisting of two major components, namely, quercetin and rutinose [10]. The presence of the four hydroxyl (-OH) groups, as well as of the rutinose component bond to the C-3 position of RUT instills this molecule with outstanding therapeutical value [12]. RUT is particularly treasured for its efficiency in counteracting strong oxidants, such as OH, superoxide, and peroxyl radicals [8]. Moreover, RUT has been found to target various signaling mediators involved in different cellular processes, such as inflammation, apoptosis, autophagy, and angiogenesis [6]. However, despite its vast applications as an active compound, RUT bears a low bioavailability owing to its hydrophobic nature, scarce affinity for cell membranes, and inefficient trans-membrane crossing, which substantially narrows its drug-likeness and use as a potential pharmaceutical [13,14]. Previous reports have shown that the derivatization of therapeutics with fatty acids ameliorates drug inconvenient properties, such as reduced lipophilicity, stability, half-life, cellular uptake, and transport through biological barriers [15]. In the case of RUT, the enzymatic acylation with hydrophobic moieties has been already established as a strategy to modify its hydrophilic/hydrophobic balance and facilitate its penetration through cell membranes, thus instilling this molecule with novel properties [16]. Aside from the physicochemical improvements, lipophilic RUT derivatives are associated with enhanced biological activities, possessing applications in various fields, such as dietetics, pharmaceuticals, and cosmetics. RUT fatty acid esters can interact with different cellular and molecular targets, exerting antioxidant, anti-tumor, vasodilatory, antiangiogenic, photo-protective, anti-aging, and anti-inflammatory properties, among other beneficial effects [17].

Based on these scientific data, the current study was designed to explore in vitro the safety and therapeutic efficiency of two rutin fatty acids derivatives, namely, rutin oleate (RUT-O) and rutin linoleate (RUT-L), as potential treatment strategies for NSCLC.

## 2. Materials and Methods

### 2.1. Specific Reagents and Instruments

The evaluated compounds, RUT-O and RUT-L, were produced through lipase-based enzymatic acylation, as described in our previous paper [10]. An MTT cell viability kit, trypsin-EDTA solution, phosphate buffer saline (PBS), dimethyl sulfoxide (DMSO), Leibovitz (L-15) medium, Trypan blue solution 0.4%, DAPI, neutral red, rutin (RUT), oleic acid (OA), linoleic acid (LA), and Triton X-100 were ordered from Sigma Aldrich (St. Louis, MO, USA), Merck KgaA (Darmstadt, Germany). The specific cell culture medium (RPMI-1640—30-2001™), FBS (fetal bovine serum), and trypsin-EDTA solution were bought from ATCC (American Type Cell Collection, Lomianki, Poland). The CyQUANT™ LDH cytotoxicity assay, Texas Red™-X phalloidin dye, alpha tubulin monoclonal antibody (B-5-1-2), and the goat anti-mouse IgG (H+L) secondary antibody (Alexa Fluor™ 488) were provided by Thermo Fisher Scientific Inc. (Waltham, MA, USA). The bovine serum albumin (BSA) was bought from Cell Signaling Technology (Danvers, MA, USA), and a paraformaldehyde 4% solution in PBS was purchased from Santa Cruz Biotechnology (Dallas, TX, USA). A penicillin–streptomycin mixture (100 U/mL–100 µg/mL) was obtained from PanBiotech (Aidenbach, Germany). EpiAiway™ 3D reconstructed human tissues were bought from MatTek Life Science Company (Bratislava, Slovak Republic). A ROS-Glo™ H_2_O_2_ assay was received from Promega Corporation (Madison, WI, USA). The Cytation 5 (plate reader), Lionheart FX (automated microscope), and Gen5™ Microplate Data Collection and Analysis Software (Version 3.14) were provided by BioTek Instruments Inc. (Winooski, VT, USA). The Olympus IX73 inverted microscope and the cellSens Dimensions v.1.8. Software were acquired from Olympus (Tokyo, Japan).

### 2.2. Safety Profile Assessment Using the EpiAirway™ 3D Model

To assess the biosafety profile of RUT-O and RUT-L (25, 50, 100, 125 µM), an MTT test was employed following a 24 h treatment of the EpiAirway™ human 3D respiratory tissue model. The protocol steps were performed according to the manufacturer’s recommendations. Briefly, immediately upon receipt, the inserts were removed from agarose, wiped using a sterile gauze, carefully transferred in 6-well plates containing 1 mL of the specific medium (AIR-100-MM) in each well, and equilibrated at 37 °C and 5% CO_2_ for 16–18 h. After this equilibration period, the cell medium was replaced with a fresh one (1 mL/well), the apical surface of the inserts was washed two times with TEER-Buffer, the tissues were treated with RUT, OA, LA, RUT-O and RUT-L solutions (50 µL sample/insert) and incubated for 24 h. In the end, the tissues were washed with TEER-Buffer, placed in a 24-well plate pre-filled with 300 µL/well of MTT solution, and incubated for 90 min. The extraction was performed by introducing the inserts in another 24-well plate containing MTT extractant solution. The absorbance was read at 570 and 650 nm, respectively, and the viabilities of the treated tissues were determined by applying a formula previously described in a publication by Moacă et al. [18].

### 2.3. Two-Dimensional Cell Culture Conditions

This study was conducted using the NCI-H23 (CRL-5800™) cell line received as a frozen vial. The NCI-H23 cells were grown in RPMI-1640 medium, supplemented with 10% FBS, and 1% antibiotics, and maintained in standard conditions for cell culture (37 °C, 5% CO_2_) in a humidified incubator, presenting normal morphology and proliferation during all the experiments.

### 2.4. Cell Viability Assessment—The MTT Test

The cell viability was assessed at the end of a 24 h treatment with RUT, OA, LA, RUT-O and RUT-L (25, 50, 100, 125 µM) by performing the MTT test according to the following steps: (i) the cells’ seeding in flat-bottom 96-well plates at a density of 10^4^ cells/well; (ii) the cells’ treatment with the investigated compounds; (iii) the addition of 10 µL MTT reagent in each well and 3 h incubation of the plates at 37 °C and 5% CO_2_; (iv) the addition of 100 µL solubilizing solution and 30 min incubation of the plates at room temperature; and (v) absorbance measurement at 570 and 630 nm. The protocol was followed as also presented by Gag et al. [19].

### 2.5. Kinetic Cell Proliferation Monitoring

The kinetic evaluation of the NCI-H23 cells’ proliferation throughout 24 h with and without the RUT-O and RUT-L (25, 50, 100, 125 µM) treatments was assessed using the instrumentation provided from Biotek (Lionheart FX microscope, and Gen5™ Microplate Data Collection and Analysis Software Version 3.14) and according to the manufacturer’s indications. The cells were seeded in flat-bottom 96-well plates in their specific culture media. After attachment, the cells were treated with the compounds of interest, the media were changed to Leibovitz’s (L-15) CO_2_-independent medium, and the plates were incubated at 37 °C in the humidity chamber of the Lionheart FX microscope. Representative images (in high contrast bright field) were taken by the instrument every hour for 24 h and analyzed to generate the cell number in each well at the set time points (from 0 h to 24 h). The results were presented by extrapolating the cell number for the control and each treatment to these time intervals.

### 2.6. Lactate Dehydrogenase (LDH) Release Quantification

The cytotoxic potential of RUT-O and RUT-L (25, 50, 100, 125 µM) was assessed by quantifying the release of the cytosolic LDH in the culture medium after 24 h of treatment. Briefly, the cells were cultured in flat-bottom 96-well plates (10^4^ cells in each well) and stimulated with RUT derivatives. At the end of the treatment interval, 50 µL of culture medium (containing released LDH) were removed from each well, transferred in new 96-well plates, and mixed with 50 µL reaction mixture. After the incubation of the plates for 30 min, 50 µL of stop solution were added, and the absorbance was read at 490 nm and 680 nm.

### 2.7. Cell Morphology Evaluation

To evaluate the influence of RUT-O and RUT-L (25, 50, 100, 125 µM) 24 h treatment on cellular morphology, the NCI-H23 cells were cultured in 12-well flat-bottom plates and imaged using the Olympus IX73 inverted microscope and the cellSens Dimensions v.1.8. software at magnification 20×, after the stimulation period.

### 2.8. Neutral Red Staining and Uptake

The protocol for this assay and the specific reagents were prepared according to Repetto et al. [20]. In brief, the cells were seeded in flat-bottom 96-well plates (10^4^ cells in each well) and treated with RUT-O and RUT-L (25, 50, 100, 125 µM) for 24 h. After the treatment, the culture medium was replaced with 100 µL/well neutral red medium (40 µg/mL neutral red solution in the specific RPMI-7951 medium for NCI-H23 cells) and incubated for 2 h at 37 °C and 5% CO_2_. Next, the cells were washed with 150 µL/well PBS and imaged in color brightfield with a Lionheart FX automated microscope. The final steps consisted of adding 150 µL neutral red destain solution (a mixture of 50% ethanol 96%, 49% deionized ultrapure water, and 1% glacial acetic acid) in each well, shaking the plates for 10 min to extract the neutral red from the cells, and absorbance reading at 540 nm using Cytation 5.

### 2.9. Immunofluorescence-Based Examination of Cellular Components

The influence of RUT-L (25, 50, 100, 125 µM) on the aspect of the cell nuclei and cytoplasmic distribution of actin and tubulin fibers was evaluated by staining these cellular components with specific antibodies and dyes. For this experiment, the cells were cultured in clear-bottom, black 96-well plates; exposed to RUT-L for 24 h; fixed with paraformaldehyde 4% in PBS; and permeabilized with Triton X 0.1%; treated with a blocking solution of bovine serum albumin (BSA) 1%, alpha tubulin monoclonal antibody (B-5-1-2) (4 h at room temperature), goat anti-mouse IgG (H+L) secondary antibody (Alexa Fluor™ 488) (45 min at room temperature), TexasRed phalloidin solution (30 min at room temperature), and DAPI solution (5 min at room temperature). Each step was preceded by the cells’ washing with PBS. Finally, the cells were imaged and analyzed using the Lionheart FX automated microscope and the Gen5™ Microplate Data Collection and Analysis Software (Version 3.14).

### 2.10. ROS Production Measurement

The ROS production was evaluated after the NCI-H23 cells’ treatment with RUT-L (50, 100, 125 µM) using the ROS-Glo™ H_2_O_2_ assay. In brief, the cells were cultured in flat-bottom, white, opaque 96-well plates (10^4^ cells in each well) and treated with the test compound for 18 h. For the final 6 h of treatment, the H_2_O_2_ substrate solution was added to the wells (final volume 100 µL). Next, the prepared ROS-Glo™ Detection Solution was added, the plates were incubated for 20 min at room temperature, and the luminescence was recorded on Cytation 5. These steps are thoroughly presented in the manufacturer’s protocol.

## 3. Results

### 3.1. RUT-O and RUT-L Lack Cytotoxicity in EpiAirway™ 3D Tissues

**Figure 1:**
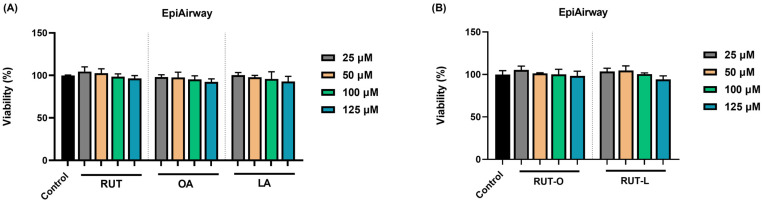
Viability of EpiAirway™ 3D human tissues after 24 h of exposure to (**A**) the parent compounds rutin (RUT), oleic acid (OA), and linoleic acid (LA), as well as to (**B**) their derivatives, rutin oleate (RUT-O) and rutin linoleate (RUT-L), at 25, 50, 100, and 125 µM. The results were presented as viability percentages (%) normalized to control (non-treated cells) and expressed as means ± standard deviation of three experiments performed in triplicate. The statistical differences between the control and the treated groups were analyzed using the one-way ANOVA and Dunnett’s multiple comparisons tests.

To investigate the potential cytotoxicity of the obtained derivatives and their parent compounds at the pulmonary level, the impact of RUT, OA, LA, RUT-O, and RUT-L (at concentrations ranging between 25 and 125 µM) on the viability of EpiAirway™ 3D human tissues was assessed following a 24 h incubation. The obtained results, graphically represented in Figure 1, indicate that the tested compounds caused no significant impairment in the EpiAirway™ tissues’ viability. Despite being reduced by RUT, OA, and LA in a concentration-dependent manner, the viability percentage maintained around 90% at the highest tested concentration. In the case of RUT-O and RUT-L, at the lowest concentrations (25 and 50 µM), both derivatives slightly elevated the viability percentages that reached values over 100%. At 100 µM, the viability of the inserts remained similar to the control for both RUT-O and RUT-L, while at 125 µM, the compounds reduced their viability to 98% and 94%, respectively.

### 3.2. RUT-L Significantly Reduces the Viability of NCI-H23 Cells

**Figure 2:**
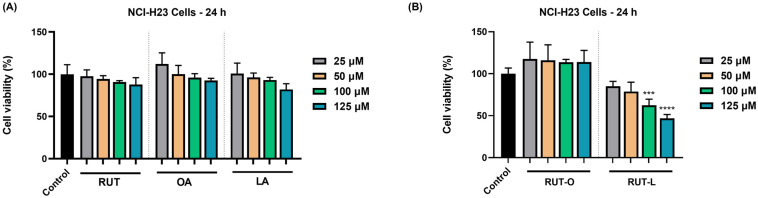
Graphical representation of the percentage of viable NCI-H23 cells after 24 h of treatment with (**A**) the parent compounds rutin (RUT), oleic acid (OA), and linoleic acid (LA), as well as with (**B**) their derivatives, rutin oleate (RUT-O) and rutin linoleate (RUT-L), at 25, 50, 100, and 125 µM. The results were presented as viability percentages (%) normalized to control (non-treated cells) and expressed as means ± standard deviation of three experiments performed in triplicate. The statistical differences between the control and the treated groups were analyzed using the one-way ANOVA and Dunnett’s multiple comparisons tests (*** *p* < 0.001; **** *p* < 0.0001).

The potential impact of RUT, OA, LA, RUT-O, and RUT-L (25, 50, 100, and 125 µM) on the viability of NCI-H23 cells was explored at the end of 24 h of treatment. As revealed in Figure 2, the parent compounds caused a dose-dependent but statistically insignificant reduction in the NCI-H23 cells’ viability. The OA at 25 µM also showed a slight stimulatory activity, the viability increasing over 100%. A clear distinction between the cytotoxic profiles of RUT-O and RUT-L was obtained at this stimulation interval, with the cell viability percentages varying depending on the tested compound and concentration. Thus, RUT-O exerted no anti-tumor activity on this NSCLC cell line; on the contrary, a stimulatory effect was noticed at all concentrations, with the viability values rating between 117% (at the lowest concentration—25 µM) and 113% (at the highest concentration—125 µM). RUT-L, on the other hand, induced a concentration-dependent reduction in the viability of the NCI-H23 cells, the lowest and most significant values being obtained at the highest tested concentrations (100 µM—63% and 125 µM—47%).

### 3.3. RUT-L Inhibits the Proliferation of NCI-H23 Cells

**Figure 3:**
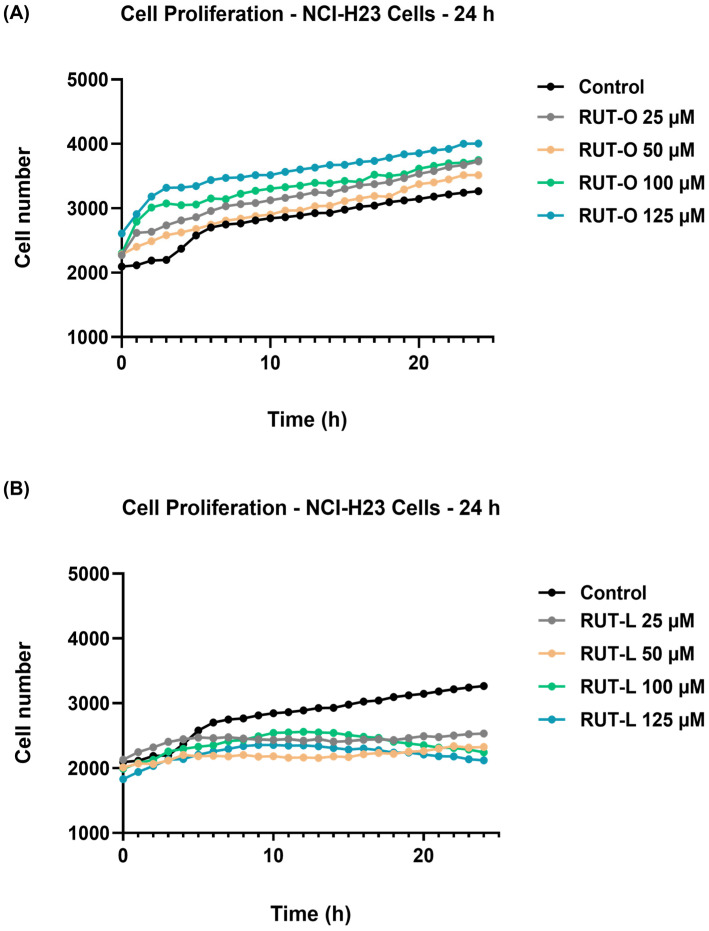
Graphical representation of NCI-H23 cells’ proliferation profile with and without exposure to (**A**) rutin oleate (RUT-O) and (**B**) rutin linoleate (RUT-L) at 25, 50, 100, and 125 µM. Cell number was measured every hour over 24 h.

Further, the potential influence of RUT-O and RUT-L (25, 50, 100, and 125 µM) on the proliferation of NCI-H23 cells was kinetically monitored every h for a period of 24 h. The obtained results are presented in Figure 3. As expected, the control (non-treated) cells presented normal proliferation during this period, with the cells’ number continuously increasing from 0 h to 24 h. A curve elevation was obtained at 4 h, suggesting a significant increase in cell proliferation starting at this time point. The highest number of control cells was quantified at the end of the measurement (24 h). The RUT-O treatment (Figure 3A) showed a stimulatory effect on cell proliferation by increasing their number at all concentrations and time intervals. In the case of RUT-L (Figure 3B), however, a different effect was observed. At 25 and 50 µM, the cell number increased continuously from 0 h to 6 h; however, from this point to 24 h, it remained relatively constant, which stresses an inhibitory effect on the cells’ proliferating ability. Finally, following the treatment with RUT-L 100 and 125 µM, the number of NCI-H23 cells increased constantly up to 12 h and gradually dropped until the end of the treatment, with the lowest values in cell number being obtained at these concentrations, highlighting the bioconjugate’s cytotoxic potential.

### 3.4. RUT-L Significantly Increases LDH Release from NCI-H23 Cells

**Figure 4:**
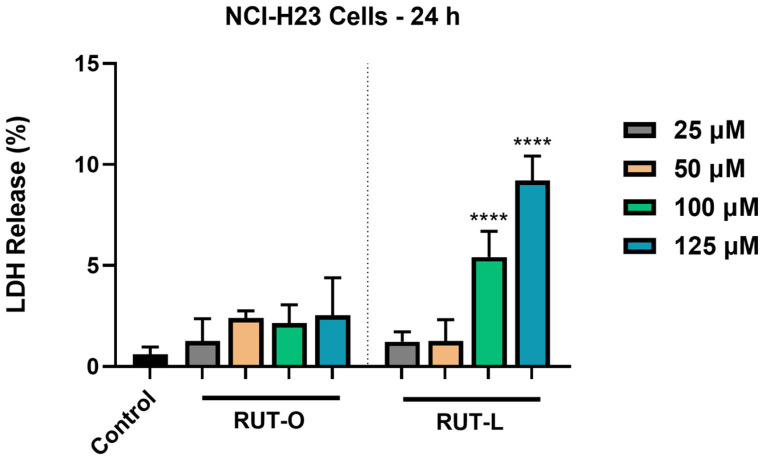
Lactate dehydrogenase (LDH) release percentages in NCI-H23 cells post-exposure to rutin oleate (RUT-O) and rutin linoleate (RUT-L) at 25, 50, 100, and 125 µM for an interval of 24 h. The results were expressed as means ± standard deviation of three experiments performed in triplicate. The statistical differences between the control and the RUT-O- and RUT-L-treated groups were analyzed using the one-way ANOVA and Dunnett’s multiple comparisons tests (**** *p* < 0.0001).

The impact of RUT-O and RUT-L (25, 50, 100, and 125 µM) on cell membrane integrity in the NCI-H23 cells was appraised by quantifying the leakage percentage of LDH in the extracellular environment at the end of the 24 h treatment. As shown in Figure 4, both derivatives had elevated LDH release compared to the control. RUT-O led to increases in the LDH release in the culture medium at all the tested concentrations, although the values were not statistically significant when compared to the non-treated cells. Significant leakage of LDH from the cytoplasmic area was obtained after the NCI-H23 cells’ exposure to RUT-L 100 and 125 µM, the calculated percentages at these concentrations being 5% and 9%, respectively.

### 3.5. RUT-L Triggers Cytoplasmic Vacuolation in NCI-H23 Cells

**Figure 5:**
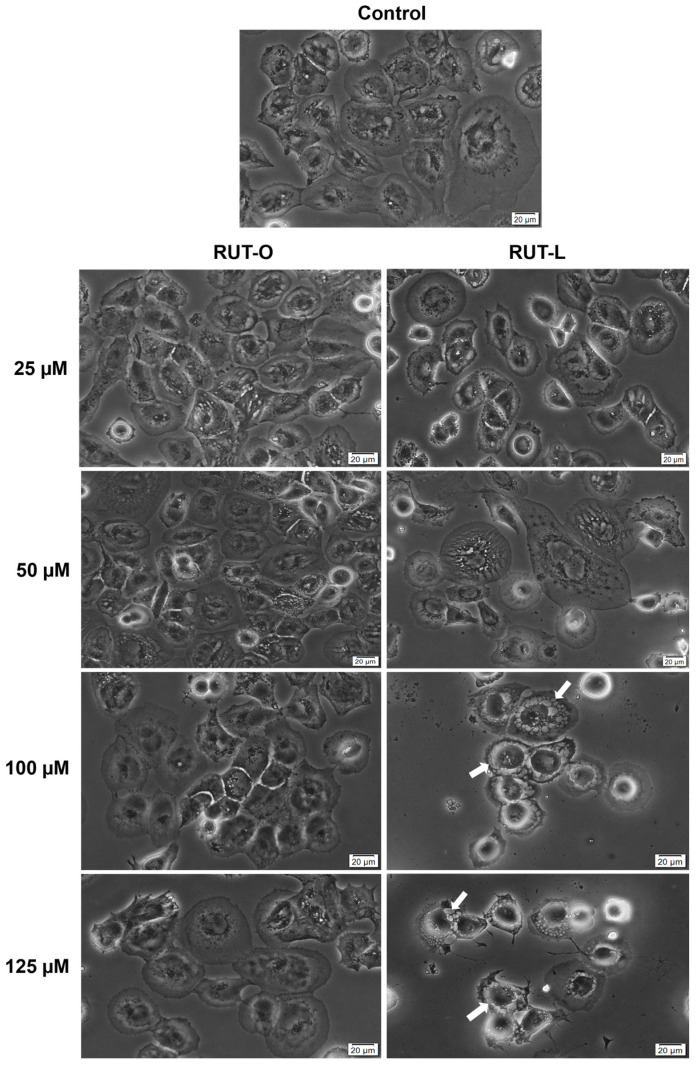
Representative images illustrating the morphology and confluence of NCI-H23 cells post-treatment with rutin oleate (RUT-O) and rutin linoleate (RUT-L) at 25, 50, 100, and 125 µM for an interval of 24 h. The white arrows indicate cells presenting cytoplasmic vacuolation. The scale bars indicate 20 µm.

To assess whether the morphology of NCI-H23 cells undergoes any changes following their treatment with RUT-O and RUT-L, a microscopic evaluation was performed (Figure 5). RUT-O showed no significant changes in the NCI-H23 cells’ morphology compared to the control at the tested concentrations. RUT-L triggered a dose-dependent decrease in cell confluence starting from the concentration of 25 µM, which was more prominent at 100 and 125 µM and was accompanied by several cytotoxic signs (i.e., cell shrinkage, rounding, and detachment). An interesting change in the cells’ appearance was observed after their 24 h treatment with RUT-L at high concentrations (100 and 125 µM), namely, cytoplasmic vacuolation (white arrows), evidenced by the formation of numerous irregular vesicles in the cytoplasm that covered the perinuclear space. At the highest tested concentration (125 µM), the cytoplasmic vacuolation was also associated with massive cell shrinkage.

### 3.6. RUT-L Blocks Neutral Red Uptake in NCI-H23 Cells

**Figure 6:**
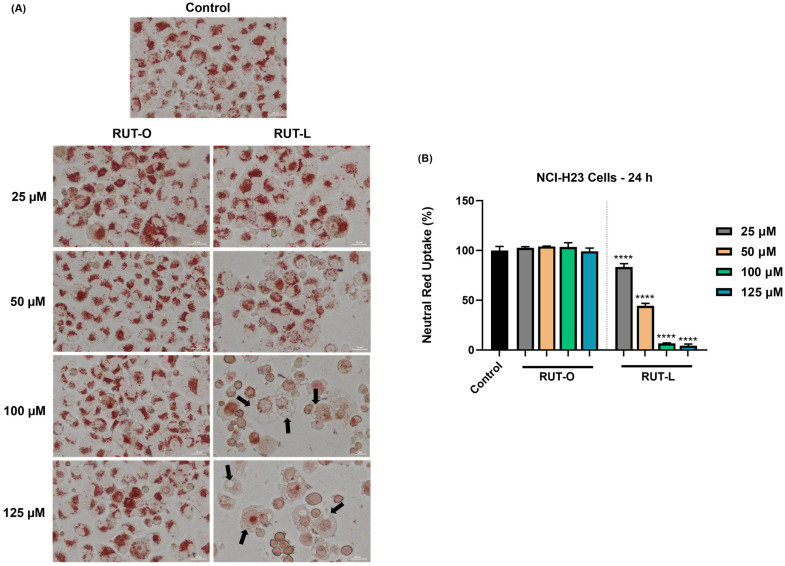
(**A**) Representative images showing the internalization of neutral red dye in NCI-H23 cells treated with rutin oleate (RUT-O) and rutin linoleate (RUT-L) at 25, 50, 100, and 125 µM for 24 h. The scale bars indicate 30 µm, and the arrows show cells presenting cytoplasmic vacuolation. (**B**) Neutral red uptake percentages in NCI-H23 cells post-exposure to rutin oleate (RUT-O) and rutin linoleate (RUT-L) at 25, 50, 100, and 125 µM for an interval of 24 h. The results were normalized to control (non-treated cells) and expressed as means ± standard deviation of three experiments performed in triplicate. The statistical differences between the control and the RUT-O and RUT-L treated groups were analyzed using the one-way ANOVA and Dunnett’s multiple comparisons tests (**** *p* < 0.0001).

Considering the formation of vesicles within the cytoplasm of the NCI-H23 cells exposed to RUT-L, further investigation regarding the uptake of neutral red dye was conducted. As imaged in Figure 6, the control and RUT-O-treated cells present a uniform uptake of neutral red dye, while changes were detected only in the case of RUT-L. Thus, at the lowest concentrations (25 and 50 µM), although a reduction in cell number occurred, the neutral red remained uniformly distributed within the cells. At 100 and 125 µM, red coloration of the cells was also observed; however, the neutral red dye seemed unable to penetrate the vacuoles formed within the cytoplasm, which remain unstained. Graphically, the percentage of neutral red uptake in the cells treated with RUT-O remains similar to the control, while significant decreases were obtained for RUT-L at all concentrations: 25 µM—83%; 50 µM—44%; 100 µM—7%; 125 µM—4%.

### 3.7. RUT-L Alters Nuclear and Cytoskeletal Appearance in NCI-H23 Cells

**Figure 7:**
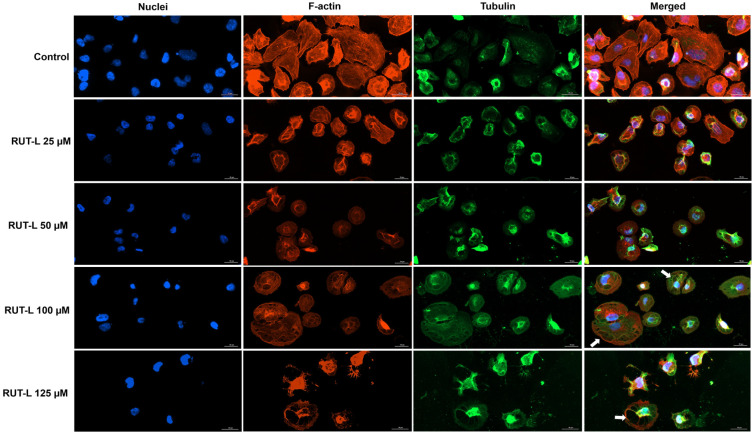
Representative images showing the aspect of nuclei, F-actin, and tubulin of NCI-H23 cells post-treatment with rutin linoleate (RUT-L) at 25, 50, 100, and 125 µM for an interval of 24 h. The scale bars indicate 30 µm, and the arrows show cells presenting cytoplasmic vacuolation.

The exposure of NCI-H23 cells to RUT-L for 24 h led to several changes in the aspect of the cell nuclei, F-actin, and tubulin. As presented in Figure 7, in the control cells, the nuclei present an oval shape and evenly distributed chromatin, while the F-actin and tubulin are uniformly distributed within the cells’ cytoplasm, emphasizing the normal epithelial-like morphology of NCI-H23 cells. The impact of RUT-L was highly dependent on the tested concentrations; however, all the treatments induced modifications compared to the control. At 25 and 50 µM, fragmentation of the cell nuclei, as well as the condensation of F-actin in the central, perinuclear area of the cells, and constriction of tubulin were observed. At 100 µM, the cytoskeletal actin and tubulin were not condensed; however, the presence of cytoplasmic vacuoles was revealed (white arrows). The nuclei presented no significant morphological changes compared to the control. At 125 µM, the aspect of chromatin, F-actin, and tubulin highlighted massive cell shrinkage. The vacuolation process, indicated by hollow spaces within the cytoplasm, is also visible at this concentration, but without nuclear fragmentation or chromatin, actin, and tubulin constriction.

### 3.8. RUT-L Generates Oxidative Stress in NCI-H23 Cells

**Figure 8:**
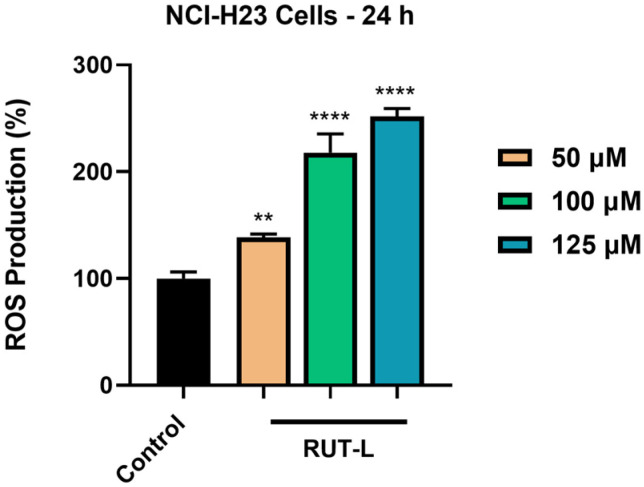
Reactive oxygen species (ROS) production (%) in NCI-H23 cells post-exposure to rutin linoleate (RUT-L) at 50, 100, and 125 µM for an interval of 24 h. The results were normalized to control (non-treated cells) and expressed as means ± standard deviation of three experiments performed in triplicate. The statistical differences between the control and the RUT-L-treated groups were analyzed using the one-way ANOVA and Dunnett’s multiple comparisons tests (** *p* < 0.001; **** *p* < 0.0001).

Finally, the ability of RUT-L, at high concentrations, to generate oxidative stress in the NCI-H23 cells after 24 h of treatment was evaluated. Figure 8 shows an elevation in the percentage of ROS in the cells exposed to RUT-L compared to the control, which increased in a concentration-dependent manner. Thus, the lowest increase was measured for RUT-L 50 µM (139%), and the highest (and almost double compared to 50 µM) for RUT-L 125 µM (252%). At 100 µM, RUT-L increased ROS generation to 218%.

## 4. Discussion

LC has become one of the most pressing health problems worldwide and a leading cause of cancer-related deaths [21]. NSCLC is the most frequently diagnosed subtype, accounting for over 80% of all LC cases. The last few decades have witnessed numerous advances made in NSCLC comprehension, leading to early disease detection and therapy development (e.g., thoracoscopic surgery, chemotherapy, radiotherapy, targeted therapy, and immunotherapy) [22]. Despite this valuable progress, the NSCLC prognosis remains poor, as the majority of the patients already present an advanced disease stage or metastases upon presentation and the current therapy options usually result in acquired resistance [21,22]. RUT is a unique flavonoid chemical possessing numerous pharmacological effects, such as antioxidant, anti-inflammatory, anti-metastatic, and anti-tumor activities [23]; its potential use as an active compound in cancer therapy is being extensively investigated at present. However, the innate low bioavailability and inconvenient pharmacokinetics of RUT [13,14] hinder its application in chemotherapy.

Thus, based on these data and with the aim of discovering new, efficient RUT-based treatments for NSCLC, the present study was conducted to explore in vitro the potential therapeutic properties of two rutin fatty acids derivatives (RUT-O and RUT-L) as improved pharmaceuticals with applications in oncology. The study was broadly stratified into three main parts: (1) the portrayal of the safety profile of RUT-O and RUT-L at the level of the lower respiratory tract, (2) the comparative assessment of the anti-tumor properties retained by RUT-O and RUT-L, and (3) the elucidation of the potential underlying mechanisms of the cytopathic effects caused by RUT-L. The results obtained in this work for RUT-O and RUT-L, at concentrations up to 125 µM, after a 24 h treatment, stress the following novelties: (1) RUT-O and RUT-L present high in vitro biocompatibility on the EpiAirway™ 3D tissues; (2) among the two tested compounds, only RUT-L exerted anti-tumor properties in NCI-H23 cells, while RUT-O showed a stimulatory effect; and (3) RUT-L induced a non-apoptotic cell death type in NCI-H23 cells, characterized by reduced cell viability, proliferation, and neutral red uptake, as well as cytoplasmic vacuolation and increased oxidative stress.

Regarding the safety of RUT-O and RUT-L for their application as potential drugs, this work stands as a continuation of a previous study [10] that described their in vitro and in ovo toxicological profile using three healthy cell lines (H9c2(2-1) cardiomyoblasts, HepaRG hepatocytes, HaCaT keratinocytes), EpiDerm™ 3D reconstructed tissues, and chick embryo chorioallantoic membrane. In terms of cytotoxicity, RUT-L showed increased in vitro safety by inducing no alterations in the cells’ viability at concentrations up to 100 µM after 24 h of treatment, whereas RUT-O reduced their viability to around 80%. Comparatively, the viability of these cell lines following their exposure to RUT, OA, and LA was maintained over 70% at the tested concentrations (1–100 µM). Both derivatives lacked irritant potential on the EpiDerm™ 3D reconstructed tissues and the chick embryo chorioallantoic membrane at the highest tested concentration (100 µM). Herein, considering the orientation of the study towards respiratory pathologies, the safety profile of these derivatives was investigated using the advanced EpiAirway™ tissues (Figure 1) at four concentrations of interest, 25, 50, 100, and 125 µM, after 24 h of treatment. EpiAirway™ represents a ready-to-use in vitro 3D model containing human tracheal and bronchial cells arranged in well-differentiated cultures that exhibit the properties and functions of in vivo tracheobronchial tissue. It presents numerous applications for acute and long-term studies in various research areas, such as infectious diseases, toxicology, pharmacology, or drug delivery [24,25]. Both RUT-O and RUT-L lacked toxicity potential in this EpiAirway™ model, with the viability of the inserts maintaining over 90% following their exposure to these bioconjugates at all the tested concentrations. A similar effect was also observed in the case of their parent compounds, RUT, OA, and LA.

The study continued with the evaluation of the compounds’ cytotoxic effects at the same concentrations in a tumorigenic cell line, NCI-H23, used as an in vitro model for NSCLC. The NCI-H23 cells, also simply named H23, present an epithelial-like morphology and applications in toxicity evaluations and cancer research, being derived from a patient diagnosed with lung adenocarcinoma [26]. The two bioconjugates showed opposite effects on the NCI-H23 cells after 24 h of treatment, with RUT-O stimulating their viability and proliferation at all concentrations, while RUT-L exerted an inhibitory effect on both cellular processes (Figure 2 and Figure 3). A possible explanation for these results might be related to the structural features of the obtained derivatives, the difference standing in the unsaturation degree of the fatty acid used for derivatization; OA is a monounsaturated fatty acid with a double bond at C9, while LA is a polyunsaturated fatty acid containing two double bonds at C9 and C12, respectively [27]. Previous publications reported the correlation between the number of unsaturated bonds in the fatty acid’s structure and its biological effects [28], as well as the higher physiological relevance and medicinal properties of fatty acids containing multiple double bonds [29].

The most significant impairments on the NCI-H23 cells’ viability were obtained at 100 and 125 µM, concentrations at which RUT-L reduced the percentages of viable cells to 63% and 47%, respectively. Based on the ISO Standard 10993-5:2009 suggesting that a compound exerts cytotoxic effects if it reduces cell viability by more than 30% [30], RUT-L can be categorized as cytotoxic at these concentrations on NCI-H23 cells whose viability was lowered under 70%. A similar effect on cell proliferation was observed as well, with RUT-L (100 and 125 µM) initially increasing the cell number up to 12 h and decreasing it gradually until the 24 h time interval, behavior that suggests its cytotoxic potential. RUT, OA, and LA induced no significant viability impairments in the NCI-H23 cells at the tested concentrations after 24 h of treatment. Previous research studies addressed the exploration of RUT as a potential strategy in LC treatment. Such an example would be the study conducted by Hoai and collaborators showing that RUT at concentrations of 150 and 300 µM caused cell death rates of 4.65% and 4.4% in A549 cells, while exerting a stronger effect when incorporated in a nanoemulsion [31]. Sghaier et al. showed that RUT efficiently inhibited the viability, adhesion, and migration of A549 cells that expressed higher resistance to the flavonoid treatment (IC_50_ = 559.83 ± 3.5 µM) compared to other cancer-type cell lines [32]. As for the tested fatty acids, a previous study reported that OA and LA reduced the viability of five cancer cell lines (HT29, HCT116, HepG2, DLD1, and AGS) following a 48 h treatment, but with higher efficiency in the case of HT29 cells (IC_50_ values of 87.43 mM and 36.61 mM, respectively) [33].

The impact of RUT-O and RUT-L (25, 50, 100, and 125 µM, 24 h of treatment) on the integrity of the NCI-H23 cell membranes was next evaluated by measuring the leakage of lactate dehydrogenase (LDH), a tetrameric cytoplasmic protein released in the extracellular medium upon membrane damage. The quantification of the extracellular release of LDH also serves as an indicator of changes occurring within the cells’ membrane permeability, being previously applied for this purpose [34]. It was found that both derivatives increase LDH leakage from the cells following treatment compared to the control (Figure 4). The increase in LDH release in the extracellular media after the NCI-H23 cells’ treatment with RUT-O contradicts the results obtained in the viability and proliferation assays that illustrate its stimulatory effect. However, the higher amount of LDH compared to the control might be related not to the compound’s cytotoxicity, but to its increased lipophilicity, which enabled better crossing through biological barriers and affected membrane permeation. Regarding RUT-L, significant LDH release was obtained at 100 and 125 µM, which confirms the results obtained for cell viability and proliferation and could be related to its lipophilic nature, as well. Our previous computational estimations regarding their properties indicated a higher lipophilicity (cLogP) and lower solubility for RUT-O and RUT-L compared to their parent compound RUT, which is in agreement with the data presented in the literature [10]. A similar correlation between the modification of compounds with fatty acids, their hydrophobicity, and ability to disrupt cell membranes was found in a study conducted by Han and collaborators [35]. Viskupicova et al. showed that the conjugation of RUT with fatty acids leads to compounds with increased hydrophobicity and solubility in fats, and presented several works indicating that RUT lipophilic derivatives present better bioavailability and crossing through biological membranes [36]. A previous study revealed that the fatty esters of phloridzin with stearic, oleic, linoleic, and α-linolenic acids at the concentration of 100 µM induced significant leakage of LDH in HepG2 cells, which was higher compared to their parent compound [37].

This study further proceeded to a microscopic evaluation of the NCI-H23 cells after 24 h exposure to RUT-O and RUT-L to identify the specific morphological features of cytotoxicity (Figure 5). RUT-O caused no morphology impairments, the treated NCI-H23 cells’ shape and aspect being very similar to the control, data that support the previous results indicating its lack of cytotoxic effect in this cell line. However, significant changes in the cells’ aspect were observed following their treatment with RUT-L at all the concentrations, such as gradual loss of confluence, cell rounding, and detachment. At 100 and 125 µM, RUT-L triggered the formation of vacuoles that presented an increased and irregular size, covering the cells’ cytoplasm. This phenomenon, presented in the literature as cytoplasmic vacuolation or vacuolization, represents a morphological event occurring in mammalian cells following their exposure to microbial agents (i.e., bacteria, viruses), as well as to natural or synthetic compounds. Cytoplasmic vacuolation was classified as transient and irreversible, the latter type being associated with cytopathic effects and, consequently, cell death. Besides acidic organelles, vacuolation affects other cellular components, such as the endoplasmic reticulum and the Golgi apparatus, and is usually associated with caspase-independent cell death types such as paraptosis, methuosis, necroptosis, or oncosis [38]. The specific aspect of the formed vacuoles that surrounded the cell nuclei and presented an uneven size indicated that the cytotoxicity caused by RUT-L at high concentrations might be due to paraptosis, a programmed and cell death machinery characterized by cytoplasmic vacuolation, dilatation of endoplasmic reticulum or mitochondria, increased ROS production, rupture of the plasma membrane, and lack of apoptosis-specific features, such as nuclear fragmentation, chromatin condensation, and caspase activation. Several compounds of natural origin (e.g., taxol, curcumin, hesperidin, etc.) were previously found to induce paraptosis in human cancer cells [39,40,41]. Regarding the description of paraptosis in previous studies, the formation of vacuoles presenting a similar aspect to the ones imaged in the present study was reported by Chen et al., who highlighted the ability of curcuminoid B63 to cause paraptosis-like cell death through ROS mediation in gastric cancer cells [42], and Kessel D, who characterized the paraptosis process determined by photodynamic therapy [43].

To investigate the observed formation of cytoplasmic vacuoles, the ability of the NCI-H23 cells to uptake neutral red after their treatment with RUT-O and RUT-L was studied (Figure 6). Neutral red represents a cationic phenazine dye that is considered a vital stain with multiple applications in biology, and is extensively used for cytotoxicity evaluations, as well as staining of lysosomes and vacuoles [44,45,46]. According to the results, neutral red accumulated in the RUT-O-treated cells similarly to the control, indicating its lack of influence on cell viability. In the RUT-L-exposed cells, a lower neutral red uptake compared to the non-treated cells was obtained at all the tested concentrations, which can be correlated to the viability results. However, in this assay, lower percentages were obtained, especially at 100 and 125 µM, compared to the MTT test, which might be due to the accumulation of cytoplasmic vacuoles that impaired neutral red incorporation in the intracellular space. As observed in the images from Figure 6A (for RUT-L 100 and 125 µM), neutral red was unable to penetrate through the formed cytoplasmic vesicles, which remained unstained. In its unprotonated form, neutral red crosses the plasma membrane through non-ionic passive diffusion, is protonated, and accumulates in acidic compartments [44,45], which indicates that the pH of the formed cytoplasmic vacuoles is neutral or basic.

Next, the impact of RUT-L on the aspect of actin and tubulin, which play key dynamic functions in migration, proliferation, cell death, and the response to external stimuli [47], was investigated (Figure 7). The nuclear morphology was also evaluated. The 24 h treatment of the cells with RUT-L caused significant damage in the morphology of the nuclei and cytoskeletal fibers compared to the control, the effects being dependent on the tested concentration. At 25 and 50 µM, RUT-L caused fragmentation of the cell nuclei and F-actin and tubulin condensation. At 100 and 125 µM, the presence of cytoplasmic vacuoles was detected, and the cells that exhibited vacuolation lacked nuclear, F-actin, or tubulin constriction compared to the control. At the highest concentration, cell shrinkage resulting from the massive constriction of all the stained cellular components was also observed.

Finally, we assessed whether the production of ROS was related to the observed cytotoxic effects induced by RUT-L. As shown in Figure 8, the increase in ROS was highly dependent on the tested concentration, the highest percentages being detected at 100 and 125 µM (218% and 252%, respectively). The ability of active phytocompounds to exert pro-oxidant activity at high doses was previously reported [48]. In the case of flavonoids, the cytocidal effects leading to the activation of cellular death pathways in malignant cells were attributed to their efficiency in causing excessive oxidative stress [49]. A concentration-dependent increase in ROS production was also obtained after the treatment of Caski cervical cancer cells with RUT (90, 120, 150 µM) for 12 h [50].

## 5. Conclusions

Aiming to develop novel chemotherapeutics for NSCLC treatment through the derivatization of RUT with fatty acids and assess their therapeutic potential in vitro, the present work portrays the lack of cytotoxicity in EpiAirway™ 3D reconstructed tissues and distinctive effects on NCI-H23 cells of RUT-O and RUT-L. Among the two tested conjugates, only RUT-L possessed anti-cancer efficiency in vitro by significantly reducing cell viability and proliferation, triggering LDH leakage, inducing cytoplasmic vacuolation and cytoskeletal deformations, reducing neutral red uptake, and causing oxidative stress. These results describe the differential therapeutic properties retained by RUT lipophilic derivatives and the potential implication of RUT-L in non-apoptotic cell death pathways, such as paraptosis.

## Data Availability

The presented data are available on request from the corresponding author.
